# A Case of Solid-Appearing Struma Ovarii: Pitfall in the Assessment Using Ovarian-Adnexal Reporting and Data System Magnetic Resonance Imaging Score

**DOI:** 10.7759/cureus.58176

**Published:** 2024-04-13

**Authors:** Takenori Isozaki, Mitsuru Matsuki, Akane Yamamoto, Suzuyo Takahashi, Harushi Mori

**Affiliations:** 1 Radiology, Jichi Medical University, School of Medicine, Tochigi, JPN; 2 Radiology, Jichi Children's Medical Center, Tochigi, JPN; 3 Obstetrics and Gynecology, Jichi Medical University, School of Medicine, Tochigi, JPN

**Keywords:** dermoid cyst, o-rads, benign, ovarian tumor, struma ovarii

## Abstract

Struma ovarii is a monodermal teratoma characterized by the presence of >50% thyroid tissue. It is mostly benign; therefore, preoperative diagnosis is important. It usually manifests as a multilocular cystic mass but rarely as a predominantly solid mass. On magnetic resonance imaging (MRI), solid-appearing struma ovarii showed early signal intensity enhancement on dynamic gadolinium-enhanced T1-weighted images, which histopathologically indicates the presence of thyroid tissue with abundant blood vessels.

The Ovarian-Adnexal Reporting and Data System (O-RADS) MRI score is a validated classification worldwide for characterizing adnexal lesions. Based on the morphology, signal intensity, and enhancement of any solid tissue on the MRI, the scoring system can be used to classify adnexal lesions into five categories from score one (no adnexal mass) to score five (high risk of malignancy). An adnexal solid mass with a higher signal intensity than that of the myometrium 30-40 seconds after gadolinium (Gd) injection on non-dynamic contrast-enhanced (non-DCE) MRI was assigned a score of 5 (high risk of malignancy).

We present a case of solid-appearing struma ovarii with a higher signal intensity than that of the myometrium 30 seconds after Gd injection on non-DCE MRI, and it was classified as score five preoperatively. Therefore, a total abdominal hysterectomy with bilateral salpingo-oophorectomy was performed despite the presence of a benign ovarian mass. When an adnexal mass with a higher signal intensity than that of the myometrium 30-40 seconds after Gd injection on non-DCE MRI is encountered, struma ovarii should be included in the differential diagnosis, despite the O-RADS MRI score of five and management of the situation should be discussed.

## Introduction

Struma ovarii is a rare monodermal variant of ovarian teratoma accounting for only 2-4% of all mature teratomas [1-2]. Histopathologically, it is defined by the presence of thyroid tissue comprising more than 50% of the overall mass [3]. Malignant transformation is rare, found in <5% of patients with struma ovarii [4]. The median age at diagnosis is between 40 and 60 years [5], and the typical clinical symptom is a non-specific palpable pelvic mass. There is no known specific tumor marker.

The typical imaging finding is multilocular cystic mass, and the characteristic findings are loculi with high density on unenhanced computed tomography (CT) and low signal intensity on T2-weighted images (T2WI) within the mass, reflecting thyroid colloid follicles with high viscosity [6]. Rarely does it appear a predominantly solid mass with early enhancement on dynamic gadolinium-enhanced T1-weighted images (Gd-T1WI) [7].

We experienced a case of struma ovarii with a solid appearance. The mass was enhanced more intensely than myometrium 30 seconds after gadolinium (Gd) injection on non-dynamic contrast-enhanced (non-DCE) magnetic resonance imaging (MRI). Therefore, the mass was evaluated as high risk of malignancy (score of 5) by the Ovarian-Adnexal Reporting and Data System (O-RADS) MRI score and total abdominal hysterectomy with bilateral salpingo-oophorectomy (TAH-BSO) was performed in spite of benign ovarian mass. We report the pitfalls in the assessment of struma ovarii with a solid appearance by O-RADS MRI score.

## Case presentation

**Figure 1:**
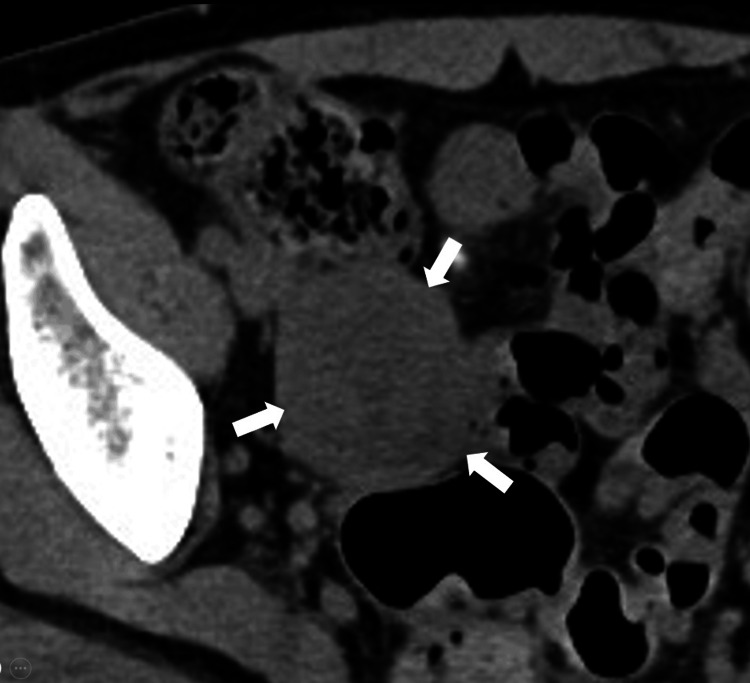
Unenhanced computed tomography (CT) image of the right adnexal mass. Unenhanced CT for systemic screening showed a solid mass in the right adnexa with 4.0 × 3.6 cm diameter and low density in comparison to the skeletal muscle.

A 47-year-old woman was referred to the hospital for further examination of an enlarged thyroid gland. She was perimenopausal with no prior pregnancies. Thyroid function tests (free T3, free T4, and TSH) were within normal range. Tumor markers (CEA, CA125, SCC, CA19-9, and AFP) were within normal range. A transrectal ultrasound imaging showed a solid hypoechoic tumor in the right adnexa. Unenhanced CT for systemic screening indicated a solid mass in the right adnexa with 4.0 × 3.6 cm in diameter and low density in comparison to the skeletal muscle (Figure 1).

**Figure 2:**
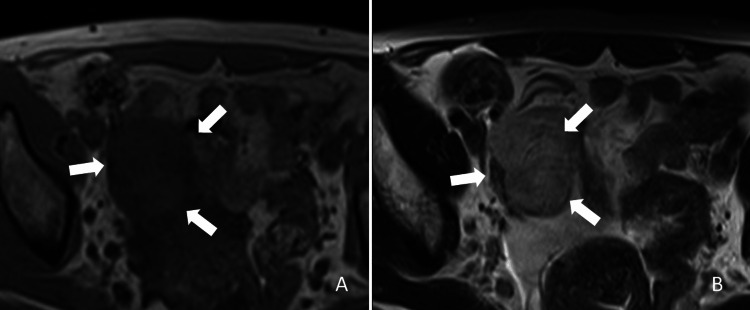
Magnetic resonance images, (A) T1-weighted image, and (B) T2-weighted image. The right adnexal mass shows an iso-signal intensity on the T1-weighted image (A, arrows) and heterogeneously high signal intensity (B, arrows) on the T2-weighted image in comparison to the skeletal muscle.

On MRI, the mass shows an iso-signal intensity on T1WI and a heterogeneously high signal intensity on T2WI compared to skeletal muscle (Figure 2).

**Figure 3:**
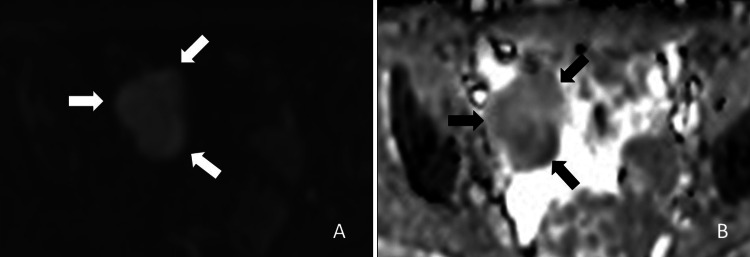
Magnetic resonance images, (A) diffusion-weighted image, and (B) apparent diffusion coefficient map. The adnexal mass shows mildly high signal intensity on the diffusion-weighted image (b = 1000 s/mm^2^) and heterogeneously high signal intensity on the apparent diffusion coefficient (ADC) map (ADC value = 1.12 × 10^-3^ mm^2^/s).

The mass shows mildly high signal intensity on diffusion-weighted images (DWI) and heterogeneously high signal intensity on the apparent diffusion coefficient (ADC) map (ADC value = 1.12 × 10^-3^ mm^2^/sec) (Figure 3).

**Figure 4:**
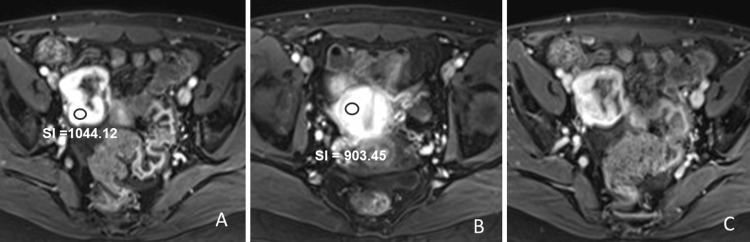
Non-dynamic contrast-enhanced T1-weighted images (A, B) 30 seconds after the gadolinium injection, and (C) 120 seconds after the gadolinium injection. On non-dynamic contrast-enhanced T1-weighted images, the adnexal mass (A, signal intensity = 1044.12) is enhanced more intensely than the myometrium (B, signal intensity = 903.45) 30 seconds after the gadolinium injection. The mass is enhanced with an internal poorly enhanced area 120 seconds after the gadolinium injection (C).

According to non-DCE MRI, postcontrast T1WI was obtained 30 and 120 sec after the Gd injection. After 30 seconds after the Gd injection, the adnexal mass was enhanced more intensely than the myometrium and determined to score 5 (high risk of malignancy) (Figure 4).

**Figure 5:**
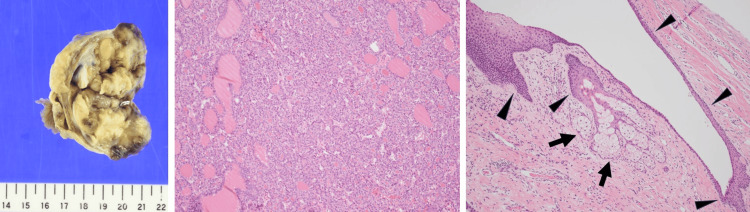
Histopathological findings. The gross specimen shows a grayish-white, solid mass on the cut surface. Histopathology (hematoxylin-eosin stain, low-power field) shows thyroid tissue with small follicles. Stratified squamous epithelium (arrows) and sebaceous glands (arrowheads) are found in the periphery.

Preoperatively, a malignant adnexal tumor was suspected. She did not wish to preserve her fertility, TAH-BSO was planned. Intraoperative frozen-section histopathology suggested a diagnosis of struma ovarii or strumal carcinoid with malignant potential. Therefore, TAH-BSO was performed. Postoperative histopathology revealed a struma ovarii consisting mainly of thyroid tissue with a few sebaceous glands and stratified squamous epithelium, and a diagnosis of struma ovarii was made (Figure 5).

## Discussion

Struma ovarii is usually shown as a well-defined multilocular cystic mass on imaging. It may show high-density loculi within the mass on unenhanced CT, which reflect iodine in thyroid colloid follicles [8-9]. Shen et al. reported CT values of struma ovarii in 12 cases ranging from 16.2-98.0 HU, in which eight cases (66.7%) had CT values of 58 HU or higher [10]. It may show calcification of the thickened septum or cyst wall. MRI shows low signal intensity loculi within the mass on T2WI in 62% of cases, which reflect thyroid colloid follicles with high viscosity [6]. Fatty component is found in 46% of cases [6]. On Gd-T1WI, thickened cyst walls, septations, and solid components show intense enhancement, which reflects thyroid tissue with abundant vessels [6]. On dynamic contrast-enhanced T1WI, the solid component shows early enhancement, followed by a plateau, and may show a steeper rise than the myometrium in the initial phase. The differential diagnosis of early enhanced adnexal mass on contrast-enhanced T1WI like in this case includes generally various benign and malignant tumors such as struma ovarii and sclerosing stromal tumors, high-grade serous carcinoma, clear cell carcinoma, endometrial carcinoma, carcinoid tumor, granulosa cell carcinoma, and Krukenberg's tumor.

MRI plays an important role in the evaluation of adnexal masses. Recently, the American College of Radiology (ACR) O-RADS MRI committee published a lexicon and risk stratification system for adnexal lesions. This scoring system includes morphology, signal intensity, and enhancement of any solid tissue. This scoring system uses a curve plotted from dynamic contrast-enhanced (DCE) MRI with a minimal temporal resolution of 15 seconds. If DCE MRI is not feasible, T1WI at 30 to 40 seconds after contrast injection can be performed instead, which is classified as non-DCE MRI in O-RADS MRI [11]. O-RADS MRI score is evaluated on a score of 1 to 5. Score 1, which is defined as no lesion (or functional cyst), score 2 as <0.5% risk of malignancy, score 3 as <5% risk of malignancy, score 4 as about 50% risk of malignancy, and score 5 as about 90% risk of malignancy [9]. Scores 4 or 5 are suggestive of malignancy and have been reported to have a sensitivity of 0.93 and 0.92, respectively, and a specificity of 0.91 and 0.90, respectively, in skilled and junior readers [12]. On DCE MRI, a contrast pattern in which the contrast effect of the enhancement component is lower than that of the myometrium, with progressive contrast and no shoulder is considered low risk, a pattern with early enhancement and a shoulder that is lower than that of the myometrium is considered intermediate risk, and a pattern with early enhancement that is higher than that of the myometrium is considered high risk [11]. Like in this case, on non-DCE MRI, an adnexal solid mass with enhancing more intensely than myometrium 30-40 seconds after Gd injection is assigned a score of 5 (high risk of malignancy) [13].

## Conclusions

In conclusion, we present a case of struma ovarii with a solid appearance with more intense enhancement than myometrium 30 seconds after gadolinium (Gd) injection on non-dynamic contrast-enhanced (non-DCE) MRI, which was classified as Ovarian-Adnexal Reporting and Data System (O-RADS) MRI score 5 (high risk of malignancy) preoperatively. Therefore, a total abdominal hysterectomy with bilateral salpingo-oophorectomy (TAH-BSO) was performed in spite of a benign ovarian mass. In encountering an adnexal mass with more intense enhancement than myometrium 30-40 seconds after Gd injection on non-DCE MRI, struma ovarii should be included in the differential diagnosis regardless of the O-RADS MRI score 5 and management should be discussed.
